# Effects of High-Definition and Conventional Transcranial Direct-Current Stimulation on Motor Learning in Children

**DOI:** 10.3389/fnins.2018.00787

**Published:** 2018-10-31

**Authors:** Lauran Cole, Adrianna Giuffre, Patrick Ciechanski, Helen L. Carlson, Ephrem Zewdie, Hsing-Ching Kuo, Adam Kirton

**Affiliations:** ^1^Department of Neurosciences, University of Calgary, Calgary, AB, Canada; ^2^Cumming School of Medicine, University of Calgary, Calgary, AB, Canada; ^3^Hotchkiss Brain Institute, University of Calgary, Calgary, AB, Canada; ^4^Department of Pediatrics, University of Calgary, Calgary, AB, Canada; ^5^Alberta Children’s Hospital Research Institute, University of Calgary, Calgary, AB, Canada; ^6^Department of Clinical Neurosciences, University of Calgary, Calgary, AB, Canada

**Keywords:** tDCS, HD-tDCS, motor learning, non-invasive brain stimulation, developmental neuroplasticity, child

## Abstract

**Background:** Transcranial direct current stimulation (tDCS) can improve motor learning in children. High-definition approaches (HD-tDCS) have not been examined in children.

**Objectives/Hypothesis:** We hypothesized that primary motor cortex HD-tDCS would enhance motor learning but be inferior to tDCS in children.

**Methods:** Twenty-four children were recruited for a randomized, sham-controlled, double-blinded interventional trial (NCT03193580, clinicaltrials.gov/ct2/show/NCT03193580) to receive (1) right hemisphere (contralateral) primary motor cortex (M1) 1 mA anodal conventional 1 × 1 tDCS (tDCS), (2) right M1 1 mA anodal 4 × 1 HD-tDCS (HD-tDCS), or (3) sham. Over five consecutive days, participants trained their left hand using the Purdue Pegboard Test (PPT_L_). The Jebsen–Taylor Test, Serial Reaction Time Task, and right hand and bimanual PPT were also tested at baseline, post-training, and 6-week retention time (RT).

**Results:** Both the tDCS and HD-tDCS groups demonstrated enhanced motor learning compared to sham with effects maintained at 6 weeks. Effect sizes were moderate-to-large for tDCS and HD-tDCS groups at the end of day 4 (Cohen’s *d* tDCS = 0.960, HD-tDCS = 0.766) and day 5 (tDCS = 0.655, HD-tDCS = 0.851). Enhanced motor learning effects were also seen in the untrained hand. HD-tDCS was well tolerated and safe with no adverse effects.

**Conclusion:** HD-tDCS and tDCS can enhance motor learning in children. Further exploration is indicated to advance rehabilitation therapies for children with motor disabilities such as cerebral palsy.

**Clinical Trial Registration:** clinicaltrials.gov, identifier NCT03193580.

## Introduction

Transcranial direct stimulation (tDCS), a form of non-invasive brain stimulation, has potential to modulate cortical excitability, human brain function, and behavior. Such promise has advanced studies across diverse brain disorders with over 33,000 sessions completed (Bikson et al., 2016). Safety and tolerability are well defined but mechanisms are poorly understood. Both preclinical and human evidence suggests long-term potentiation (LTP)-like mechanisms are involved (Stagg and Nitsche, 2011). As is often the case with emerging therapeutics, pediatric populations have been understudied in tDCS research where <2% of studies have been dedicated to the developing brain.

The primary motor cortex (M1), a key structure in motor skill learning, can be purposefully modulated with brain stimulation (Reis et al., 2008). tDCS animal models have demonstrated how polarizing, subthreshold direct currents can alter cortical excitability, neuronal firing and the size of evoked potentials (Bindman et al., 1964). tDCS over M1 increases cortical excitability (Nitsche and Paulus, 2000) and, when paired with training of the contralateral hand, improves motor performance within single (Nitsche et al., 2003; Boggio et al., 2006; Vines et al., 2006) and multiple (Reis et al., 2009) sessions. Cathodal stimulation of the ipsilateral M1 can also improve motor skill acquisition, presumably via effects on transcallosal, interhemispheric motor networks (Fregni and Pascual-Leone, 2007). Recently, we demonstrated that such M1 tDCS approaches can enhance motor learning in healthy children over 3 days of training with retained effects and large effect sizes (Ciechanski and Kirton, 2016). Stimulation was well-tolerated with no adverse events. Limited evidence suggests tDCS- induced electric fields differ in the pediatric brain though mechanistic investigations of tDCS in pediatrics are lacking (Stagg and Nitsche, 2011; Dayan et al., 2013; Kessler et al., 2013; Moliadze et al., 2015).

High-definition tDCS (HD-tDCS) may provide more focal current delivery to better target functional cortical regions. By placing a central anode surrounded by four cathodes, 4 × 1 HD-tDCS can be applied in a more focused manner with generation of stronger regional electric fields (Dmochowski et al., 2011). HD-tDCS can increase motor adaptation within a single session (Doppelmayr et al., 2016), and bimanual hand dexterity over multiple days in adults (Pixa et al., 2017). To date, HD-tDCS has not been examined in a pediatric population.

The ease of application of tDCS has promoted its early translation toward childhood disability and CP. Perinatal stroke is the leading cause of hemiparetic CP, affecting millions worldwide with few effective treatments (Oskoui et al., 2013; Kang et al., 2016). Perinatal stroke is an ideal human model of developmental plasticity where targeting M1 has shown potential for therapeutic neuromodulation (Kirton, 2016). Although the models are different, trials in adult stroke hemiparesis suggest tDCS may facilitate motor rehabilitation (Kang et al., 2016; Elsner et al., 2017). Preliminary evidence of efficacy has been suggested in trials of contralesional tDCS in hemiparetic children (Kirton et al., 2017; Gillick et al., 2018). There is a pressing need to optimize tDCS enhancement of motor learning in pediatrics to advance such therapies and better outcomes for disabled children.

We therefore conducted a sham-controlled, double-blind, randomized trial to compare the effects of M1 tDCS and HD-tDCS on motor learning in typically developing children.

## Materials and Methods

### Trial Design and Participants

Accelerated Motor Learning in Pediatrics (AMPED) was a randomized, double-blind, single-center, sham-controlled interventional trial. The study was registered at clinicaltrials.gov (NCT03193580). This study was carried out in accordance with the recommendations of the University of Calgary Research Ethics Board with informed written consent from all participants. All participants and their guardians provided written informed consent and assent when applicable in accordance with the Declaration of Helsinki. The protocol was approved by the Conjoint Health Research Ethics Board, University of Calgary (REB16-2474).

Participants were recruited through community and school outreach programs and the Healthy Infants and Children Clinical Research Program. Inclusion criteria were: (1) age 12–18 years, (2) right handed (self/parent report and Edinburgh Handedness Inventory), (3) typical neurodevelopment, (4) no major medical conditions, and (5) informed consent/assent. Persons with neuropsychiatric diagnoses/medication or implanted devices were excluded.

**FIGURE 1:**
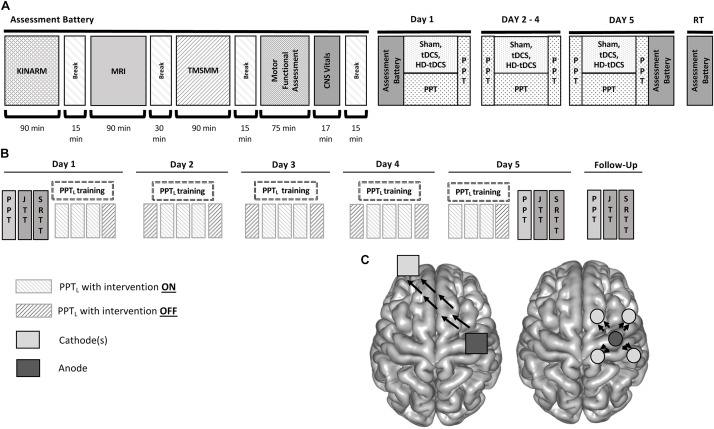
Accelerated motor learning in pediatrics (AMPED) protocol. **(A)** Participants received an MRI, complete tasks in a virtual reality KINARM robotic system, received TMS motor mapping (TMSMM), completed a series of motor assessments and then received training paired with non-invasive brain stimulation interventions. On days 2–4, subjects perform the PPT during intervention. Participants repeat the Day 1 tasks on Day 5 (with training) and at a 6-weeks retention testing follow up (RT). **(B)** PPT training is paired with stimulation by treatment groups with electrode montages **(C)** shown for tDCS (left) and HD-tDCS (right) where dark gray electrodes are anodes and light gray electrodes are cathodes. Black arrows represent the direction of current flow from anode to cathode(s).

Each participant underwent the same testing, training, and stimulation procedures over five consecutive days. The complete study design and flow is shown in Figure 1.

Previous evidence of tDCS-enhanced motor learning in children (Ciechanski and Kirton, 2016) suggested moderate-to-large effect sizes and typical means and variances (1.5- to 2-fold increases with mean standard deviation of 0.71). Combining these with α = 0.05 and a power of 85% estimated a total requirement of 24 participants (8 per stimulation group) using an online sample size calculator^1^.

### Randomization, Blinding, and Concealment

Using the Research Electronic Data Capture tool (REDCap), participants were randomized into three groups (1:1:1): (1) right M1 1 mA anodal conventional 1 × 1 tDCS (tDCS), (2) right M1 1 mA anodal 4 × 1 HD-tDCS (HD-tDCS), or (3) sham. Participants and their parents were blinded to treatment assignment consistent with previous pediatric trials (Ciechanski and Kirton, 2016). A post-interventional questionnaire asked participants to guess which intervention they received and why. Only the investigator administering the stimulation was aware of the treatment group.

### Motor Learning Measures

The primary motor learning measure was the PPT, a validated measure of hand dexterity commonly used in motor learning studies (Tiffin and Asher, 1948; Gardner and Broman, 1979). The PPT consists of four subtests, the first three require participants to place as many pegs as they can in a pegboard in 30 s with their left hand (PPT_L_), right hand (PPT_R_)_,_ and bimanually (PPT_LR_). The sum of the three scores was also generated (PPT_S_). The final subtest was a bimanual assembly task (PPT_A_). The total number of pegs was recorded. The PPT_L_ was used to train and measure motor learning as a challenging task for children to learn without reaching a skill “ceiling” effect. The PPT_R_ evaluated effects in the untrained hand. Secondary, untrained motor outcomes included the JTT which assessed six subtests of common unimanual hand functions (Elizabeth Reedman et al., 2015) and the SRTT which measured reaction time and implicit motor learning (Nissen and Bullemer, 1987; Honda et al., 1998).

### Intervention: tDCS and HD-tDCS

Participants received direct-current stimulation or sham during each training session using a conventional 1 × 1 tDCS or a 4 × 1 HD-tDCS system (Soterix Medical Inc., New York). Using the T1 images acquired from their MRI, each participant’s right M1 was localized using neuronavigation via an optical detection camera system (Brainsight2, Rouge Research Inc., Montreal; Polaris NDI Medical Solutions, Ontario, Canada). Robotic single-pulse TMS localized the “hotspot” for the first dorsal interosseous muscle of the left hand using established criteria (Zewdie and Kirton, 2016).

For tDCS and sham, two saline-soaked sponge electrodes (25 cm^2^, SNAPpad, Soterix) were applied to the scalp. The active electrode (anode) centered over the right M1 and the cathode over the contralateral supraorbital area (Figure 1C). Electrodes were held in place with a light plastic pediatric “headband” (SNAPstrap, Soterix Medical Inc., New York). The electrodes were then connected to a 1 × 1 DC SMARTscan Stimulator (Soterix). This was the same montage described in both adult and pediatric tDCS motor learning studies (Vines et al., 2006; Reis et al., 2009; Reis and Fritsch, 2011; Ciechanski and Kirton, 2016).

For HD-tDCS, the montage targeted the right M1 as described elsewhere (Caparelli-Daquer et al., 2012; Villamar et al., 2013; Richardson et al., 2015). Participants wore “cap” with pre-existing electrode holes. The anode was centered over the right M1 with four cathodes spaced ∼5 cm away to establish a ring-like orientation (Figure 1C) (12 cm diameter, Sintered ring HD-electrode, Soterix) (Datta et al., 2009; Kuo et al., 2013; Villamar et al., 2013, p. 1; Alam et al., 2014). Electrodes were then connected to a 4 × 1 HD-tDCS Adaptor and a SMARTscan Stimulator (Soterix).

During active stimulation, current was initially ramped up to 1 mA over 30 s and maintained for 20 min with automatic continuous current-control. Current was then ramped down to 0 mA over 30 s and was current-controlled based on continuous sampling of resistance. In the sham group, current was initially ramped to 1 mA over 30 s then immediately ramped down to 0 mA. During the final 30 s, the current ramped up to 1 mA and back to 0 mA. This sham procedure has been validated in subjects naïve to tDCS (Ambrus et al., 2012).

### Training Protocol

The sequence of motor measures, motor training, and stimulation are summarized in Figure 1B. Participants could complete all tasks within a single session on Day 1 (∼8 h) or split up Day 1 into two consecutive days (∼4 h each). On Day 1, baseline skill was measured by completing all motor tasks. Each participant then trained the PPT_L_ while receiving either tDCS, HD-tDCS, or sham. Training occurred over 20 min of stimulation, consisting of three trials per epoch performed at minute 5, 10, and 15. After stimulation, the electrodes were removed and participants completed a safety and tolerability questionnaire (below). The PPT_L_ was then performed again. On days 2, 3, and 4, participants performed the same PPT_L_ sequence, beginning with a baseline test followed by the same 20-min training protocol during stimulation. On Day 5, participants repeated all assessments performed on Day 1, starting with the same training protocol. Participants returned 6 ± 1 weeks later and performed the same order of assessments as Day 1.

Each assessment was video-taped and re-scored by a blinded team member for quality assurance. Learning curves generated for the PPT_L_ compared the score difference at each training point with the baseline score. Skill decay was measured by comparing the 6-week follow-up score with the Day 5 post-training score. Online effects (within-day training) were determined by comparing baseline and final scores of each day. Offline effects (consolidation) were measured by comparing baseline scores each day to the final score from the previous day.

### Safety and Tolerability

Participants completed a pediatric non-invasive brain stimulation safety and tolerability questionnaire (Garvey et al., 2001) immediately following each session (days 1–5). The duration and severity of any symptoms were reported including headache, neck pain, unpleasant tingling, itching, burning, fatigue, nausea, and lightheadedness. Participants were asked to rank the tolerability of their session compared to seven common childhood experiences. As the first study of HD-tDCS in children, a neuropsychological battery was completed at baseline and following the final stimulation session. A validated, computerized assessment (CNS Vital Signs) evaluated neurocognitive status (Gualtieri and Johnson, 2006). An interim safety analysis was conducted by two blinded researchers after the first eight subjects to exclude any drop-in motor function (reduction of PPT) of either hand or serious adverse events.

### Statistical Analysis

For the primary hypothesis, a linear mixed effect model analysis was employed (SPSS 25.0.0, IBM, Armonk, NY, United States) with fixed effects for Group, Day, and the interaction of Group and Day and random effects for Subjects including the intercept to account for repeated measures. The dependent variable for linear mixed modeling was PPTL score, independent variables being group and day. Analysis for secondary continuous outcomes (SigmaPlot 12.5, Systat Software Inc.; San Jose) included one-way analyses of variance (ANOVA) and Chi-square/Fisher’s exact test for dichotomous variables to compare group demographics, baseline motor scores, and tolerability. Paired *t*-tests analyzed differences in left and right-hand motor scores, skill decay, and online/offline effects. Effect sizes were reported as Cohen’s *d*. Repeated measures ANOVA was used to analyze changes in JTT and SRTT scores. Holm–Sidak *post hoc* corrections were performed to correct for multiple comparisons. To examine possible effects of baseline function, participants were divided into high and low performers based on baseline PPT_L_ scores above or below the median.

### Replication

To evaluate replicability of previous studies while adding to the power of the current study, we combined our data with that of a previous, similar trial (Ciechanski and Kirton, 2016). Both studies had the same inclusion criteria and applied right M1 tDCS (1 mA) or sham during 20 min of PPT_L_ training over three consecutive days. Accordingly, learning curves over 3 days of training from sham controls and anodal tDCS groups were combined (*n* = 14 for both groups). The linear mixed modeling analysis was repeated with the combined data. The fixed effects were Group, Day, and the interaction of Group and Day. The random effects were Subjects including the intercept to account for repeated measures.

## Results

### Population Characteristics

Twenty-four participants were recruited (median age 15.5 years, range 12–18, 52% female). All completed all stages and outcomes with no drop-outs. Demographics and baseline motor function across groups are shown in Table 1. Age, sex, handedness, and function did not differ between groups (all *p* > 0.3). All groups demonstrated higher PPT_R_ compared to PPT_L_ scores (*p* < 0.001).

**Table 1:** Participant demographics and baseline motor function.

				Baseline PPT scores			Baseline JTT scores				
Stimulation group	Age (years)	Laterality index	Sex F:M	Left Hand	Right Hand	Left vs. Right *p*-value	Left hand	Right hand	Left vs. Right *p*-value	Baseline reaction time excluded (ms)	Baseline reaction time included (ms)
HD-tDCS	14.77	81.25	4:4	13.91	15.79	*p* < 0.001	21.91	20.23	*p* = 0.053	540	540
	(±2.0)	(±14.7)		(±1.9)	(±1.55)	*t* = –4.97	(±2.1)	(±2.2)	*t* = 1.86	(±82.6)	(±82.6)
tDCS	15.94	82.5	3:5	13.50	15.21	*p* = 0.011	22.92	20.63	*p* < 0.001	525	525
	(±1.5)	(±13.1)		(±1.3)	(±1.9)	*t* = –2.93	(±3.1)	(±2.9)	*t* = 6.97	(±99.0)	(±98.7)
Sham	15.81	81.9	6:2	13.83	15.16	*p* = 0.013	21.09	18.92	*p* = 0.003	538	550
	(±1.3)	(±22.8)		(±1.3)	(±1.9)	*t* = –2.83	(±2.9)	(±1.9)	*t* = 3.96	(±78.5)	(±99.2)
Mean	15.51	81.88	13:11	13.75	15.4	*p* < 0.001	21.97	19.92	*p* < 0.001	540	544
	(±1.7)	(±16.6)		(±1.5)	(±1.7)		(±2.7)	(±2.4)		(±84.7)	(±91.8)
Between group	*p* = 0.324	*p* = 0.879	*p* = 0.309	*p* = 0.846	*p* = 0.741	–	*p* = 0.424	*p* = 0.342	–	*p* = 0.924	*p* = 0.859


*JTT, Jebsen–Taylor Test; PPT, Purdue Pegboard Test*.

### Motor Learning

**FIGURE 2:**
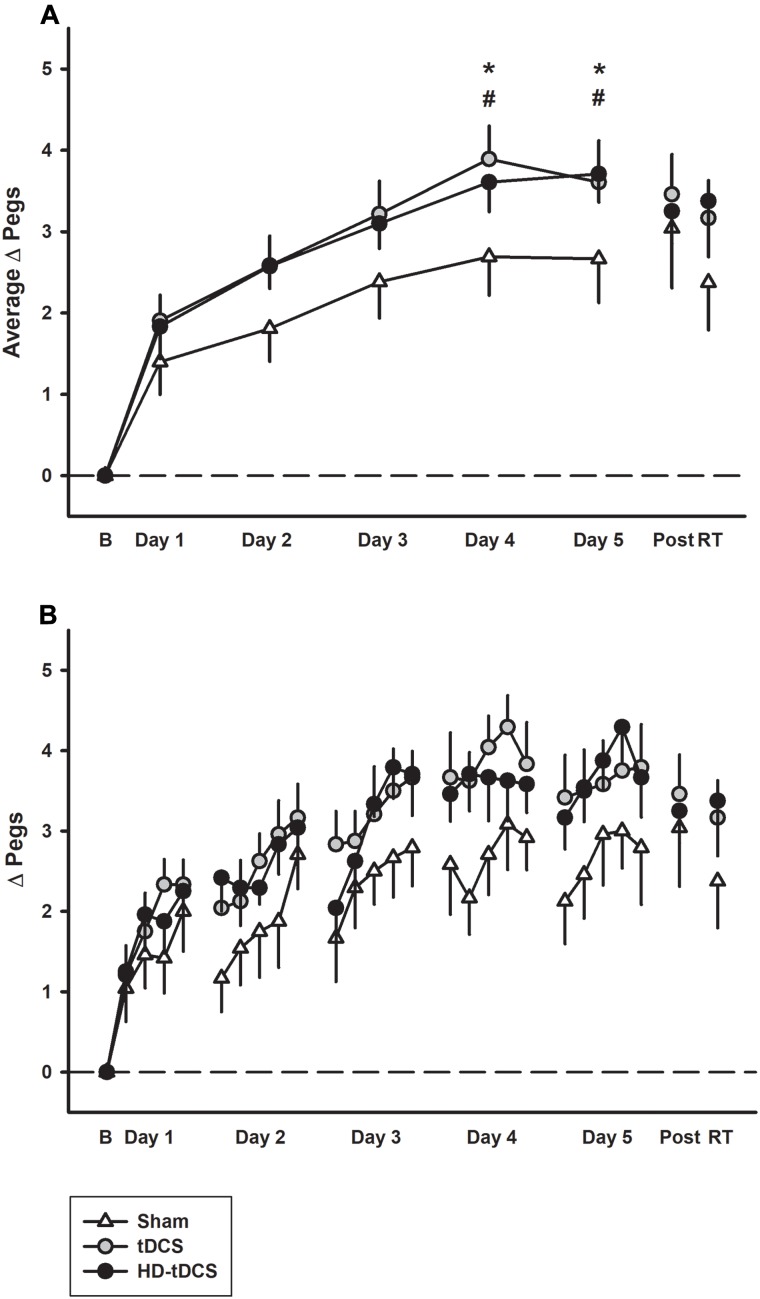
Motor learning by treatment group. **(A)** The mean daily change from baseline (B) in left Purdue Pegboard (PPT_L_) learning curves for sham (white triangles) were lower than both tDCS (gray circles) and HD-tDCS (black circles). Effects decayed by 6 weeks retention time (RT) for sham but not tDCS groups. **(B)** Daily mean scores per repetition of the PPT_L_ are shown for the same three groups. ^∗^*p* < 0.05 for tDCS vs. sham, ^#^*p* < 0.05 for HD-tDCS vs. sham.

Learning curves of similar morphology were generated across subjects and groups. Daily motor learning and daily average learning curves by group are shown in Figure 2. Regardless of intervention, participants demonstrated motor learning over the 5 days with the rate dissipating over time [*F*(2,24) = 23.7, *p* < 0.001]. Linear modeling demonstrated a significant interaction effect of day and intervention on the rate of learning. The active stimulation groups demonstrated enhanced learning (increase in pegs/day) compared to sham (tDCS *p* = 0.042, HD-tDCS *p* = 0.049). The sham group improved their PPT_L_ score by 0.508 ± 0.190 pegs/day compared to 0.703 ± 0.269 for the tDCS group and 0.697 ± 0.269 for the HD-tDCS group. At both days 4 and 5, the tDCS and HD-tDCS groups had larger improvements in the daily average PPT_L_ score compared to sham (tDCS: *p* = 0.026, *p* = 0.038; HD-tDCS: *p* = 0.043, *p* = 0.05; days 4 and 5, respectively). Moderate to large effect sizes were observed in the tDCS and HD-tDCS groups at the end of day 4 (Cohen’s *d* tDCS = 0.960, HD-tDCS = 0.766) and day 5 (Cohen’s *d* tDCS = 0.655, HD-tDCS = 0.851).

### Retention

Learning effects were retained in the tDCS and HD-tDCS groups with no decrease in skill performance between end of training and the retention assessment. In contrast, skill decay was observed in the sham group (*p* = 0.034, *t* = 2.16). At retention testing, there was no difference in PPT_L_ scores between intervention groups (*p* = 0.456).

### Online/Offline Learning

**FIGURE 3:**
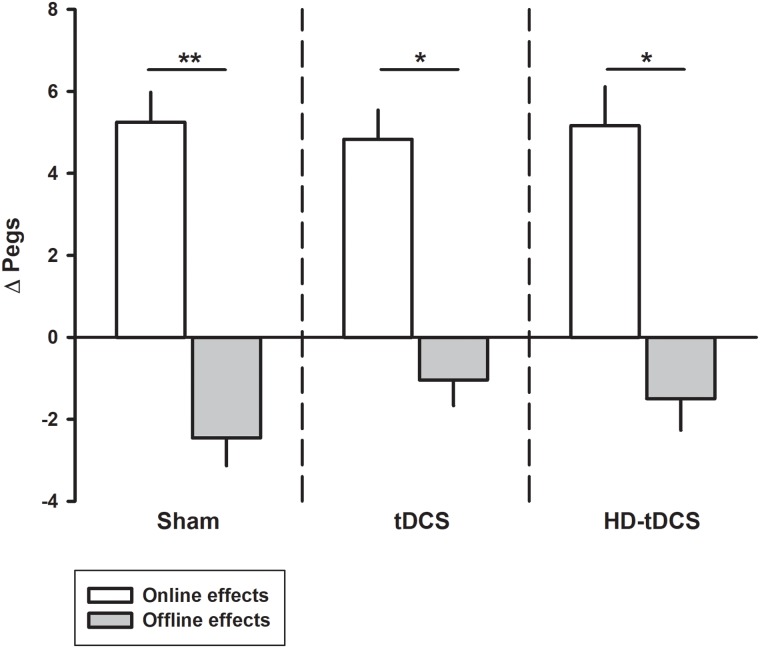
Online and offline learning effects on left hand Purdue Pegboard (PPT_L_) for the three intervention groups. The online effects represent the difference in PPT_L_ score from the first and last training point of the day. The offline learning represents the difference between the last training time point of the day to the first training point of the following day. ^∗^*p* < 0.05, ^∗∗^*p* < 0.01.

Performance improvements occurred primarily through online learning (all *p* < 0.003, *t* > 4.03; Figure 3). There were no differences seen between tDCS or HD-tDCS and sham in the amount of online learning. Offline effects contributed to a significant loss of skill in the sham and HD-tDCS group (sham *p* = 0.004, *t* = 3.64; HD-tDCS *p* = 0.046, *t* = 1.95) but not the tDCS group (*p* = 0.070, *t* = 1.67).

### Low Versus High Performers

**FIGURE 4:**
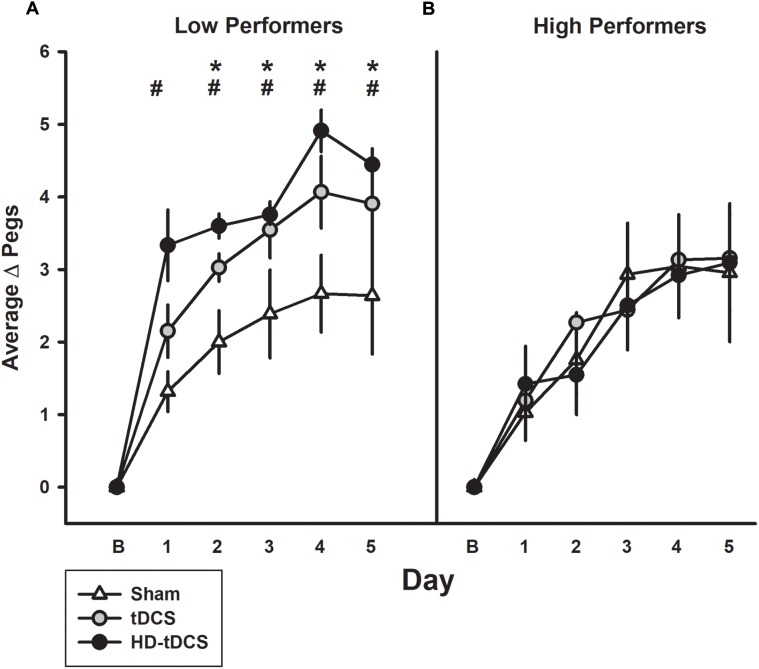
Effect of performance status on motor learning enhancement. **(A)** Low performers (baseline PPT_L_ below the median score) demonstrated marked separation of PPT_L_ learning curves with tDCS (gray circles) and HD-tDCS (black circles) outperforming sham (white triangles). **(B)** Treatment group effects were not observed for high performers. B refers to baseline. ^∗^*p* < 0.05 for sham vs. tDCS, ^#^*p* < 0.05 for sham vs. HD-tDCS.

The median baseline PPT_L_ score for all participants was 13.33 with 11 participants above this classified as high performers and the remaining participants being low performers (Figure 4). In the low performance group, the tDCS and HD-tDCS groups demonstrated greater improvements compared to sham. The high performer group did not show any difference in learning across groups.

### Secondary, Untrained Motor Outcomes

**FIGURE 5:**
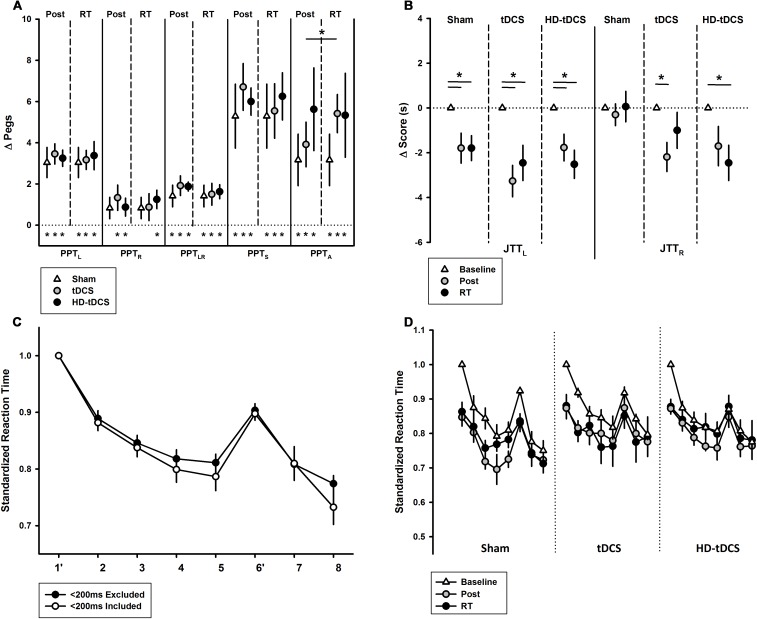
Secondary motor outcomes. **(A)** Change in Purdue Pegboard Test (PPT) scores at post-training and retention time (RT) demonstrated treatment group effects for PPT_A_. PPT subtests are left (PPT_L_), right (PPT_R_), bimanual (PPT_LR_), sum of scores (PPT_S_), and assembly (PPT_A_). ^∗^*p* < 0.05. **(B)** Jebsen–Taylor Test of Hand Function left and right (JTT_L_, JTT_R_) demonstrated treatment group effects bilaterally at post-training and RT. **(C)** Serial Reaction Time Task (SRTT) curves with and without <200 ms responses are shown. Blocks 1 and 6 are random while all others follow a 12-character sequence. **(D)** SRTT by intervention group with <200 ms responses excluded.

The effects of intervention group on the secondary motor outcomes are shown in Figure 5. PPT_R_ scores increased following training in the tDCS and HD-tDCS groups (both *p* < 0.042) but did not change in the sham group (Figure 5A, *p* = 0.076). PPT_R_ scores were not correlated with PPT_L_ improvements (*r* = 0.266, *p* = 0.208). There was no decay in PPT_R_ scores at retention testing in all groups.

PPT_LR_ scores improved in all groups: sham (*p* = 0.016, *t* = 2.69), tDCS (*p* = 0.003, *t* = 4.00), and HD-tDCS groups (*p* < 0.001, *t* = 8.62) with no decay. There was no difference between the three groups at post-training (*p* = 0.664). PPT_LR_ scores at post-training correlated with change in PPT_L_ (*r* = 0.564, *p* = 0.004). Regardless of group, participants showed improvements in PPT_S_ scores (all *p* < 0.006), again without decay. There was also an improvement in PPT_A_ for all groups (all *p* < 0.03, *t* > 2.53) and no decay. There was no difference between the intervention groups for change in PPT_A_ (*p* = 0.506). There was an improvement in PPT_A_ from post-training to retention in the tDCS group (*p* = 0.05). There was no correlation between PPT_A_ and PPT_L_ improvements (*r* = -0.032, *p* = 0.881).

Jebsen–Taylor Test performance is summarized in Figure 5B. All three treatment groups improved their JTT_L_ scores from baseline to post-training (*p* < 0.003) and baseline to retention testing (*p* < 0.019). In the untrained hand, JTT_R_ scores improved over time from baseline to post-training (*p* = 0.005) and from baseline to retention testing (*p* = 0.019). JTT_R_ scores significantly improved in the tDCS group from baseline to post-training (*p* = 0.016) and in the HD-tDCS group from baseline to retention testing (*p* = 0.026). No changes in JTT_R_ was observed in the sham group (*p* = 0.857).

The baseline SRTT curves are summarized in Figure 5C where a downward shift indicates improved reaction time. A negative correlation was observed between baseline reaction time and age (*r* = -0.488, *p* = 0.016). There was a visible downward shift in SRTT curves for all groups. There was a significant learning effect from baseline to post-training (both *p* < 0.010) and baseline to retention (both *p* < 0.009) in the sham and tDCS groups. There was no significant learning effect in the HD-tDCS group. There was no decay in reaction time in any stimulation group.

### Replication

**FIGURE 6:**
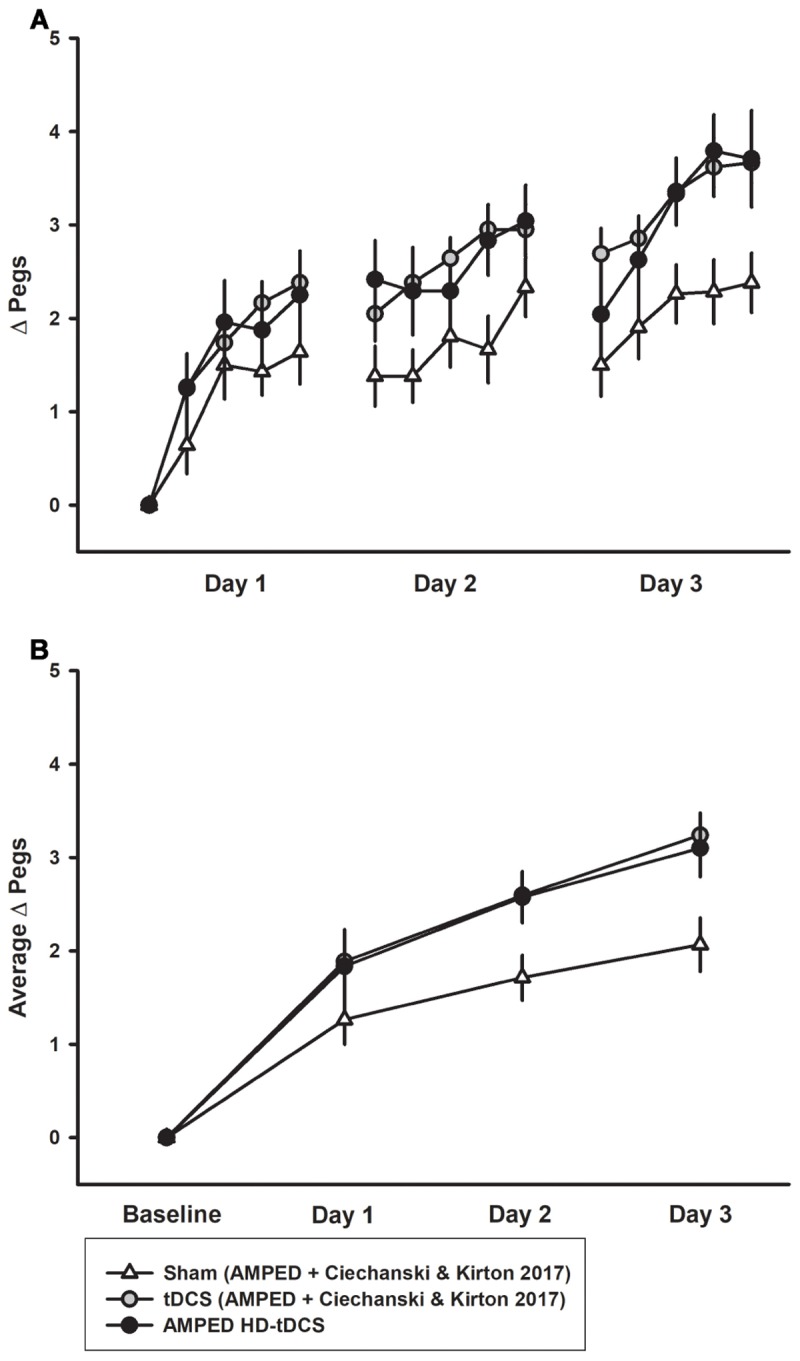
Combined PPT_L_ training data for sham and tDCS groups over 3 days. **(A)** Sham (white triangles, *n* = 14) learning curves were inferior to both tDCS (gray circles, *n* = 14) and HD-tDCS (black circles, *n* = 8) groups. **(B)** Mean daily learning for the same three groups form the combined studies. Both the tDCS and HD-tDCS groups placed more pegs each day as compared to sham.

The combined PPT_L_ dataset for 3 days of training is shown in Figure 6. There was no difference in learning between either the sham groups (*p* = 0.402) or tDCS (*p* = 0.980) groups from both studies. For the combined data, a significant interaction effect of day and intervention group (rate of learning) was shown. Effect sizes were larger with sham participants placing 0.666 ± 0.226 more pegs each day compared to 1.04 ± 0.375 for tDCS and 1.00 ± 0.320 for HD-tDCS. The tDCS and HD-tDCS group outperformed the sham group in terms of the rate of pegs placed (tDCS *p* = 0.001, HD-tDCS *p* = 0.012). Effect sizes at the end of Day 3 were large for tDCS (Cohen’s *d* = 1.265) and HD-tDCS group (Cohen’s *d* = 0.995).

### Safety and Tolerability

A total of 120 tDCS sessions were performed without any complications or serious adverse events. The most common reported sensation was itching (56%) ranging from mild (75%) to moderate (25%) in severity. Itching was more common with tDCS (78%) compared to sham (48%, *p* = 0.006) and HD-tDCS (43%, *p* = 0.001). Additional sensations included: unpleasant tingling 24% (mild in 90%) and burning 37% (mild in 80%), neither of which differed by treatment group. In the sham group, most sensations (90%) lasted less than 2 min. Sensations reported in tDCS and HD-tDCS persisted for the duration of the stimulation in 23% of tDCS and 3% of HD-tDCS sessions. One HD-tDCS participant reported a headache lasting less than 2 min. One tDCS participant felt mildly nauseated in one session. Tolerability rankings were comparable across groups: sham 3.8 ± 1.1, tDCS 4.1 ± 1.0, and HD-tDCS 3.9 ± 1.2, comparable to watching TV (2.4 ± 1.0) or a long car ride (4.9 ± 1.2). Participants were unable to correctly predict their treatment group. Baseline cognitive performance in all three domains was comparable across groups (Table 2, all *p* > 0.70). No changes in cognitive function were found for any group (all *p* > 0.07) with the single largest drop being in visual memory for the sham group.

**Table 2:** Group mean performance on the CNS Vital Signs neurocognitive battery.

Neurocognitive domain	Participant group		
Time point	Sham	tDCS	HD-tDCS
**Visual memory**			
Baseline	65.4 (20.0) [32–92]	67.4 (26.6) [18–92]	67.9 (25.2) [25–96]
Post	69.1 (19.9) [37–92]	75.0 (20.7) [45–96]	59.6 (24.7) [25–95]
Follow-up	43.5 (27.0) [7–92]	61.4 (26.3) [10–97]	65.3 (28.0) [14–88]
**Reaction time**			
Baseline	61.6 (35.0) [2–95]	53.9 (36.5) [3–95]	72.0 (17.4) [37–87]
Post	78.4 (12.3) [53–96]	61.3 (26.6) [30–96]	80.4 (18.9) [45–98]
Follow-up	68.1 (26.6) [10–96]	59.4 (34.1) [12–90]	66.4 (25.0) [14–94]
**Simple attention**			
Baseline	62.4 (19.0) [23–79]	60.4 (27.6) [13–79]	69.4 (11.1) [50–79]
Post	60.8 (19.5) [23–79]	57.5 (32.6) [2–79]	60.9 (24.2) [13–79]
Follow-up	56.4 (26.1) [1–79]	49.8 (32.0) [1–79]	73.4 (11.0) [50–79]


*Values are mean percentiles with (SD) and [range], higher values indicate better performance. tDCS, conventional anodal tDCS; HD-tDCS, high-definition tDCS*.

## Discussion

Our findings suggest that application of both conventional and HD-tDCS is feasible, safe, and well-tolerated children. The addition of tDCS or HD-tDCS of the contralateral M1 can enhance motor learning compared to training alone. Children with lower performance at baseline may be more responsive to the effects of tDCS. Skill enhancement may spill over to untrained tasks and the untrained hand.

Improvement in motor learning with tDCS has been well established in adults during both single and multi-day sessions (Reis et al., 2009; Schambra et al., 2011; Prichard et al., 2014). Despite these promising findings, pediatric studies are limited. Our findings suggest that conventional and HD-tDCS may enhance motor skill learning with retained effects. Previous studies in adults suggested that motor learning occurs mainly through online effects but tDCS enhancement acted more selectively on offline consolidation (Reis et al., 2009). Limited work in pediatric populations suggests that tDCS may enhance motor learning by modulating online systems (Ciechanski and Kirton, 2016). Our findings here may occupy a middle ground between these bodies of evidence whereby some degree of offline effect was suggested for tDCS and possibly HD-tDCS where between session decay appeared to be less pronounced as compared to sham. Extension of previous adult studies demonstrating effects of tDCS administered *after* training (Tecchio et al., 2010) have not been replicated in children and represent a potential avenue to further elucidate potential mechanisms.

Mechanisms of tDCS are difficult to study in humans with even more limited knowledge in the developing brain (Antal et al., 2003; Anguera et al., 2007; Hummel et al., 2009; Lee et al., 2010b; Biabani et al., 2018). Anodal tDCS may modulate neuronal excitability leading to increased spontaneous neuronal firing rates. Such LTP-like enhancement and strengthening of neuronal activity between stimulated and distal locations may be similar to natural motor learning processes. Human studies suggest that tDCS paired with motor training may improve the efficacy of synaptic connections with lasting effects on cortical networks (Fritsch et al., 2010). GABA systems are likely crucial in motor learning (Stagg and Nitsche, 2011) and anodal tDCS may modulate M1 GABA in adults (Stagg et al., 2011). The use of advanced imaging before and after such trials may shed further light on the mechanisms of tDCS enhanced motor learning in children.

Motor learning and stimulation effects were not limited to the trained hand. Both pediatric and adult studies have shown tDCS-related improvements in the untrained hand (Pereira et al., 2011; Ciechanski and Kirton, 2016; Hamoudi et al., 2018). We have previously shown that M1 tDCS is associated with improvements in the untrained hand and spill-over to untrained tasks (Ciechanski and Kirton, 2016). This transfer of skill was only evident in the active stimulation groups even though assessments were performed hours after stimulation when changes in neuronal excitability may still be present (Nitsche and Paulus, 2001; Moliadze et al., 2015). Improvements of the untrained hand could be secondary to various mechanisms (Anguera et al., 2007; Lee et al., 2010b). Increases in motor cortical excitability (Nitsche and Paulus, 2000), facilitation of motor performance (Antal et al., 2003; Nitsche et al., 2003; Boggio et al., 2006; Hummel et al., 2009), and potentiation of the formation of motor memories (Galea et al., 2010) have been reported after M1 tDCS. The “callosal access” and “bilateral access” hypothesis proposes that practice-induced motor engrams developed in the dominant hemisphere may be accessed in homologous regions of the opposite motor cortex (Anguera et al., 2007; Lee et al., 2010a). Others suggest the improvements reflect an increase of excitatory (or decrease of inhibitory) drive toward M1 (Biabani et al., 2018) and paired-pulse TMS studies have demonstrated suppression of intracortical inhibitory systems after tDCS (Biabani et al., 2018). MR spectroscopy studies have also shown a decrease in GABA after M1 tDCS (Stagg et al., 2014). Increases in BDNF have also been hypothesized to modulate neuroplastic potential (Biabani et al., 2018). Another possible theory invokes a role of mirror visual feedback affecting M1 plasticity (Nojima et al., 2012; von Rein et al., 2015). Mechanisms clearly remain to be defined.

The development of computational current models assists in understanding brain electric field strengths as well as potential differences in tDCS of children (Datta et al., 2009; Kessler et al., 2013). Age-related differences may include tissue structure, age-dependent differences in skull thickness, myelination, and volumes of CSF, gray matter, and white matter (Brain Development Cooperative Group, 2012). Pediatric current modeling suggests that electrodes placed on M1 produce diffuse cortical effects including contralateral M1 and bilateral premotor and supplementary motor areas (Kessler et al., 2013; Ciechanski et al., 2018). In contrast, HD-tDCS produces more focal stimulation with peak electric fields approximating functional cortical targets directly under the active electrode (Datta et al., 2009). Despite this potentially increased specificity, there have been few studies of HD-tDCS in motor learning and none in children. Improvement in motor behaviors in single (Doppelmayr et al., 2016) and multiday (Pixa et al., 2017) HD-tDCS training studies appear consistent with our findings here. Our direct comparison of conventional to HD-tDCS provides further insight, though implications for mechanism remain speculative. One simple interpretation would be that stimulation of M1 is most important for enhancing motor learning effects as both montages accomplished this, likely to a comparable degree. A different hypothesis was that the stimulation of larger areas of the motor network (e.g., premotor and supplementary motor areas) and other frontal regions (prefrontal cortex) might be mediating the previously described effect of 1 × 1 tDCS. If this was the case, we would have expected the tDCS group to outperform the HD-tDCS group. That this did not occur provides indirect but informative evidence suggesting that M1 remains a major target for motor system neuromodulation approaches. We cannot rule out, however, that these other motor regions, or other areas such as primary sensory cortex, were not involved in the tDCS effects observed here.

Our study adds novel safety data. tDCS has been applied to thousands of subjects across a diverse array of brain disorders without evidence of harm (Bikson et al., 2016). Given that the wide distribution of conventional tDCS-induced electric fields and no previous HD-tDCS studies in children, we conducted screening cognitive tests before and after intervention with no signs of change. This supports the standing premise of functional targeting; neurostimulation likely only modulates neurophysiological processes that are themselves undergoing endogenous plastic change. In our population, tDCS was well-tolerated with itching and tingling being common as reported previously (Ciechanski and Kirton, 2016). HD-tDCS had comparable sensations and tolerability to tDCS. Adjusting saline concentration may be a method to improve tolerability and decrease sensation severity in children (Dundas et al., 2007, p. 200). Increasing the separation distance between stimulation electrodes may improve the tolerability of increased scalp current with HD-tDCS (Datta et al., 2009). The inability of subjects to guess their treatment assignment supports effective blinding of accepted sham techniques.

The translational significance of our findings is evident for the 17 million people in the world living with CP, the leading cause of lifelong disability (Oskoui et al., 2013). With M1 as the primary target, four early phase clinical trials of non-invasive stimulation paired with intensive motor therapy have shown evidence of safety and possibly efficacy in children with hemiparetic CP (Gillick et al., 2016, 2018; Kirton et al., 2016, 2017). That lower baseline skill level was also predictive of responding to stimulation-enhanced motor learning here is consistent with previous results in young adults (Ciechanski et al., 2017) and also therapeutically relevant if in fact those with poor motor skills may be more responsive. Clinical trials of neurostimulation in disabled pediatric populations require special considerations (Kirton, 2016) but should be driven by the first principles established in healthy populations as presented here.

Our study was limited by our modest sample size which was evidence-based but we encountered higher variability in outcomes than anticipated. This may have decreased our ability to fully define any potential differences between tDCS and HD-tDCS. Other factors may dictate differences in motor learning and response to neurostimulation including genetics (e.g., BDNF), gender, age, past experience, and environmental influences (Fritsch et al., 2010). Fatigue is another such factor and our study protocol was intense for young participants. We took extensive measures to ensure consistency with multiple breaks but cannot exclude fatigue effects.

## Conclusion

tDCS and HD-tDCS of the primary motor cortex can enhance motor learning in healthy children over days with lasting effects. Trials of existing and emerging neurostimulation approaches are safe, feasible, and required to define and expand therapeutic potential for disabled children.

## Data Availability

All relevant data generated and analyzed for this study is included in the manuscript.

## Author Contributions

LC, AG, PC, and AK conceived study design. LC and AG performed participant recruitment. LC, AG, PC, EZ, and H-CK performed data collection. LC, AG, PC, and HC performed data analysis and drafting of the manuscript. LC, AG, PC, HC, H-CK, EZ, and AK revised the manuscript. AK obtained funding.

## Conflict of Interest Statement

The authors declare that the research was conducted in the absence of any commercial or financial relationships that could be construed as a potential conflict of interest.

## Footnotes

1 https://www.stat.ubc.ca/rollin/stats/ssize/n2.html

## Abbreviations

AMPED: accelerated motor learning in pediatrics
ANOVA: analysis of variance
BDNF: brain-derived neurotrophic factor
CP: cerebral palsy
HD-tDCS: high-definition transcranial direct current stimulation
JTT: Jebsen–Taylor Test of Hand Function
LTP: long term potentiation
M1: primary motor cortex
PPT: Purdue Pegboard Test
REDCap: Research Electronic Data Capture
SRTT: Serial Reaction Time Task
tDCS: transcranial direct current stimulation
TMS: transcranial magnetic stimulation
